# Distribution of 5-Methyltetrahydrofolate and Folic Acid Levels in Maternal and Cord Blood Serum: Longitudinal Evaluation of Japanese Pregnant Women

**DOI:** 10.3390/nu12061633

**Published:** 2020-06-01

**Authors:** Yoshinori Kubo, Hideoki Fukuoka, Terue Kawabata, Kumiko Shoji, Chisato Mori, Kenichi Sakurai, Masazumi Nishikawa, Takeshi Ohkubo, Kyoichi Oshida, Naotake Yanagisawa, Yuichiro Yamashiro

**Affiliations:** 1 Faculty of Nutrition, Kagawa Nutrition University, 3-9-21 Chiyoda, Sakado, Saitama 350-0288, Japan; kawabata@eiyo.ac.jp (T.K.); shoji.kumiko@eiyo.ac.jp (K.S.); 2 Department of Progressive DOHaD Research, Fukushima Medical University School of Medicine, 1 Hikarigaoka, Fukushima 960-1295, Japan; fukuokah@fmu.ac.jp; 3 Department of Bioenvironmental Medicine, Graduate School of Medicine, Chiba University, 1-8-1 Inohana, Chuo-ku, Chiba 260-8670, Japan; cmori@faculty.chiba-u.jp; 4 Department of Sustainable Health Science, Center for Preventive Medical Sciences, Chiba University, 1-33 Yayoi-cho, Inage-ku, Chiba 263-8522, Japan; 5 Department of Nutrition and Metabolic Medicine, Center for Preventive Medical Sciences, Chiba University, 1-33 Yayoi-cho, Inage-ku, Chiba 263-8522, Japan; sakuraik@faculty.chiba-u.jp; 6 Department of Food Management, School of Food, Agricultural and Environmental Sciences, Miyagi University, 2-2-1 Hatadate, Taihaku-ku, Sendai, Miyagi 982-0215, Japan; nishikaw@myu.ac.jp; 7 Department of Health Nutrition, Faculty of Human Sciences, Sendai Shirayuri Women’s College, Honda-Cho, Izumi-ku, Sendai, Miyagi 981-3107, Japan; t-ohkubo@sendai-shirayuri.ac.jp; 8 Research & Development Department, Taiko Pharmaceutical Co., Ltd. 2-3-3, Higashishinbash, Minato-ku, Tokyo 105-0021, Japan; kyoichi.oshida@seirogan.co.jp; 9 Medical Technology Innovation Center, Juntendo University, 2-1-1, Hongo, Bunkyo-ku, Tokyo 113-8421, Japan; n-yanagisawa@juntendo.ac.jp; 10 Probiotics Research Laboratory, Graduate School of Medicine, Juntendo University, 2-9-8-3F, Hongo, Bunkyo-ku, Tokyo 113-0033, Japan; yamasiro@juntendo.ac.jp

**Keywords:** pregnant woman, folic acid, 5-methyltetrahydrofolate, homocysteine, serum folate, cord blood, isotope dilution mass spectrometry

## Abstract

“Total” folate in blood has usually been measured to evaluate the folate status of pregnant women. However, folate is composed of many metabolites. The main substrate is 5-methyltetrahydrofolate (5-MTHF), with folic acid (FA) representing a very small component as an unmetabolized species in blood. We longitudinally evaluated 5-MTHF, FA and total homocysteine in maternal and cord blood from Japanese pregnant women. Subjects were 146 pregnant women who participated in the Chiba study of Mother and Child Health (C-MACH) prospective cohort study. Sera were obtained in early and late pregnancy, at delivery, and from cord blood. Species levels were measured by isotope-dilution mass spectrometry. Both 5-MTHF and FA levels were lower than reported levels from pregnant women in populations from countries with mandatory FA fortification. As gestational age progressed, serum 5-MTHF levels decreased, whereas serum FA levels were slightly reduced only at delivery compared to early pregnancy. A significant negative association between serum 5-MTHF and total homocysteine was shown at all examined times, but no associations with FA were evident. At delivery, cord 5-MTHF was significantly higher than maternal levels, while FA again showed no significant correlation. These results suggest that 5-MTHF is actively transported to the fetus through placental transporters and may reflect folate status during pregnancy as a physiologically important species.

## 1. Introduction

Folate is an essential micronutrient that mediates the transfer of one-carbon units and is involved in the biosynthesis of the thymidylates and purines that constitute nucleic acids, in the metabolism of some amino acids, and in methylation reactions of DNA, histone proteins and neurotransmitters [1]. Folate is essential for cell division, and is thus particularly important for fetal growth and the development of the uteroplacental organs, and folate requirements thus increase during pregnancy [2,3]. Maternal folate deficiency is associated with elevated homocysteine levels in blood and adverse pregnancy outcomes, such as congenital disorders, including neural tube defects (NTD) and pregnancy complications [1,4,5,6]. Folic acid (FA) is used as a supplement in the mandatory or voluntary fortification of certain foods. The FA contained in supplements and fortified foods is metabolized in the body to 5-methyltetrahydrofolate (5-MTHF), through the folate metabolic pathway [7]. In the cytoplasm, 5-MTHF supplies a methyl group to the homocysteine remethylation reaction for methionine synthesis, and this reaction acts to lower blood homocysteine levels [8].

A study of men and women aged between 29 and 86 years in the United States found that blood levels of 5-MTHF and FA were higher after fortification than before this measure was introduced [9]. Intervention studies have shown that when healthy adults continued to take supplements containing 400 µg/day of FA or FA-fortified foods for 5–14 weeks, FA was detected as an unmetabolized species in the blood. This is attributed to dihydrofolate reductase (DHFR), the rate-limiting enzyme in folate metabolism for reducing dihydrofolate to tetrahydrofolate, exceeding its capacity to metabolize FA [10,11,12,13]. One concern is that blood FA may have negative effects on the fetus [14,15]. Previous studies have not been consistent in the reported effects of excessive FA intake and the effects are unclear. [16]. In these studies, “total” folate in blood was measured when examining folate status in pregnant women, while blood FA levels were not measured [17]. Different folate species, such as FA and 5-MTHF, must therefore be measured separately.

Liquid chromatography-tandem mass spectrometry [18] (LC-MS/MS) is able to separately evaluate blood FA and 5-MTHF levels. In epidemiological studies of pregnant women, most reports detected FA in maternal or cord blood [19,20,21,22,23,24]. Studies of folate status in pregnant women in the United States and Germany have shown that serum or plasma 5-MTHF levels were higher in cord blood than in maternal blood [19,25], and maternal blood 5-MTHF levels were shown to correlate positively with cord blood [25], and showed a positive correlation between maternal FA levels and cord blood FA levels [22].

A study of folate status and lifestyles among pregnant women in the United States showed a negative association between 5-MTHF levels and smoking habits during pregnancy, and a positive association with folate intake (dietary folate equivalent µg/day). In a report investigating the association between folate status and preterm delivery in the United States, maternal 5-MTHF levels were negatively associated with a high incidence of preterm delivery [20]. However, these reports only conducted measurements at one time point and were not monitored longitudinally during pregnancy. Previous studies have thus been insufficient to elucidate the dynamics of the molecular species in folate metabolism during pregnancy. Furthermore, FA fortification of cereals is not mandatory in Japan, and the recommendation for folate intake according to Japanese Dietary Reference Intakes (DRIs) is lower than in Western countries [26]. Since the folate measurement data reported by other countries cannot be applied to Japanese populations, the analysis of blood FA and 5-MTHF levels in Japanese subjects will be useful for deciding folate nutritional guidelines in the future.

In this study, we aimed to measure serum 5-MTHF, FA and total homocysteine in Japanese pregnant women, using LC-MS/MS. Additionally, we investigate the longitudinal distribution of the aforementioned species and their changes with gestational age, associations with homocysteine, and relationship to maternal blood and cord blood. 

## 2. Materials and Methods 

### 2.1. Birth Cohort Study

This study was based on the Chiba study of Mother and Child Health (C-MACH), conducted at the Center for Preventive Medical Sciences, Chiba University and the Research Institute for Science and Engineering, Waseda University. C-MACH is a cohort study which aims to explore the effects of genetic and environmental factors, particularly the in-utero environment and the postnatal living environment, on the health of children [27]. This study was approved by the Biomedical Research Ethics Committee of the Graduate School of Medicine, Chiba University (ID: 451, 8 November 2013; ID: 462, 4 December 2013; ID: 502, 28 May 2014), the Ethics Review Committee for Human Genome/Gene Analysis Research, Waseda University (ID: 2013-G002 (3), 13 November 2015), and the Kagawa Nutrition University ethics review committee (ID: 67, 6 July 2016). All subjects provided informed consent for inclusion before participating in the study. The study was conducted in accordance with the Declaration of Helsinki.

### 2.2. Study Design

The study used a longitudinal design. Blood was collected in four sampling periods: maternal blood in early and late pregnancy (gestational age of 12 and 32 weeks, respectively) and at birth, and umbilical vein blood at birth. A self-administered questionnaire on lifestyle was conducted during early and late pregnancy at the same times as blood collection.

### 2.3. Subjects

C-MACH recruited healthy pregnant women under 13 weeks of pregnancy who visited Onodera Ladies Clinic and Yamaguchi Women’s Hospital in Chiba prefecture, and Aiwa hospital in Saitama prefecture, between February 2014 and June 2015. Follow-up was terminated if the subject had a miscarriage, stillbirth, withdrawal, or transfer [27]. This study included 146 pregnant women attending Aiwa Hospital, out of 434 pregnant women who participated in C-MACH.

### 2.4. Mother and Child Information

#### 2.4.1. Lifestyle Data

Information on marital status, parity, smoking habits and alcohol consumption during pregnancy, and household income were obtained from the self-administered questionnaires conducted during early and late pregnancy.

#### 2.4.2. Anthropometric Data

Pre-pregnancy body mass index (kg/m^2^) was calculated from height and pre-pregnancy weight obtained from the self-administered questionnaire conducted in early pregnancy.

#### 2.4.3. Medical Data of Mother and Infant

Information on maternal age at birth, gestational age, birth weight, birth length and sex were obtained from hospital medical records.

#### 2.4.4. FA Intake

Information of FA containing supplements and fortified foods about brand name, type, duration of use, frequency of intake and amount taken was collected from self-administered questionnaires administered during early and late pregnancy. Based on the method of a previous study [28], the average daily FA intake (µg/day) was calculated using the number of days FA taken, amount of FA products taken per day, and the serving size unit from the FA product label for the 4 weeks prior to the day of blood collection.

### 2.5. Measurement of Folate Metabolism-Related Substances in Serum

Simultaneous analysis of FA, 5MTHF, and total homocysteine was performed using the isotope-dilution mass spectrometry method [29,30].

#### 2.5.1. Blood Collection

Within 2 h after blood collection, centrifugation was performed at 1700× *g* for 10 min; 0.5 mL of the supernatant (serum) was dispensed, and stored frozen at −80 °C until measurement. 

#### 2.5.2. Sample Preparation

Fifty microliters of serum, 10 μL of internal standard, and 50 μL of 100 mg/mL of tris (2-carboxyethyl) phosphine and 140 μL of 1% (*v*/*v*) formic acid in methanol were mixed for 15 min, and the supernatant was centrifuged at 16,200× *g* for 5 min. Supernatant was passed through a 0.2-μm filter and set in a vial.

#### 2.5.3. Analytical Instruments

The liquid chromatography system was an Agilent 1200 Series (Agilent Technologies Japan, Tokyo, Japan), the ion source was a Turbo Ion Spray (Applied Biosystems SCIEX, Tokyo, Japan), and the triple quadrupole mass spectrometer was a 4000 QTRAP (Applied Biosystems SCIEX, Tokyo, Japan). Various parameters related to the ionization and detection of the standard substance of the measurement component and the corresponding internal standard substance were optimized [*m*/*z* 460.2–313.2 (5-MTHF), *m*/*z* 442.2–295.2 (FA), *m*/*z* 136.0–90.0 (homocysteine), *m*/*z* 465.2–313.2 (5-MTHF-^13^C_5_), *m*/*z* 447.2–295.2 (folic acid-^13^C_5_), *m*/*z* 140.0–93.9 (homocysteine-d_4_)]. After setting up these multiple reaction monitoring transitions, simultaneous analysis was performed in Scheduled MRM mode. The measurement time was 13 min, the mobile phase flow rate was 500 μL/min, A: perfluoroheptanoic acid 5 mM aqueous solution and B: acetonitrile gradient, and the separation column used was XSelect HSST3 2.5 μm, 100 × 2.1 (Nihon Waters, Tokyo, Japan).

#### 2.5.4. Measurement and Data Analysis

Sample measurements were performed twice, then the average value was used. Additionally, a calibration curve was created at 8 points every 24 h and quality control was conducted every 12 h. In preliminary validity tests, the coefficient of variations of FA, 5-MTHF, and total homocysteine were 9.9%, 4.7% and 4.1%, respectively, for intra-assay and 3.7%, 8.4% and 2.3% for inter-assay, respectively. Analyst version 1.6.3 analysis software (Applied Biosystems SCIEX, Tokyo, Japan) was used for data processing and quantification. If the peak could not be detected or the signal-to-noise ratio was less than 10, the concentration was converted to 0.

### 2.6. Statistical Analysis

The distributions of serum 5-MTHF, FA and total homocysteine levels used in the analysis were skewed, so continuous variables are shown as medians and interquartile ranges. The Wilcoxon signed-rank test was used to compare the folate metabolism-related substance levels in maternal serum between each blood sampling period (*n* = 113), and to compare the FA intake between early and late pregnancy (*n* = 118). Bonferroni correction was used to adjust for multiple comparisons (*p* < 0.0167). The difference between maternal blood and cord blood was tested using the Wilcoxon signed rank test (*n* = 114). Spearman’s rank correlation coefficient was used for the correlation between two variables. The significance level was *p* < 0.05 (two-tailed test). All statistical analyses were performed using JMP^®^ Pro version 12.2.0 (SAS Institute Japan, Tokyo, Japan).

## 3. Results

**Figure 1:**
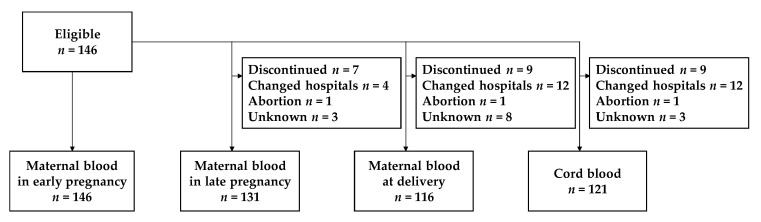
Flow of participants among pregnant women participating in Chiba study of Mother and Child Health (C-MACH).

Figure 1 shows the participant flowchart for final number of blood sample analysis at each time point. At the time of recruitment, 146 samples could be measured in early pregnancy, but further serum samples could not be obtained from some subjects, with 131 samples obtained in late pregnancy, 116 at delivery, and 121 from cord blood. 

Table 1 shows the characteristics of mothers who provided valid responses to the lifestyle questionnaire. All participants in the cohort were Japanese. Mean age (±standard deviation) of the mother at birth was 32.3 ± 4.6 years. Most subjects were married and did not smoke or drink during pregnancy. The proportion of pregnant women who took FA in early pregnancy was 54.6%; this decreased to 32.5% in late pregnancy. FA intake (µg/day) in late pregnancy was significantly lower than in early pregnancy (*p* < 0.0001, *n* = 118).

Table 2 shows the characteristics of the neonates. The preterm birth rate was 1.7%. Mean birth weight was 3155 ± 369 g, and the percentage of low birth weight infants was 3.3%.

Table 3 shows the distribution of serum 5-MTHF, FA and total homocysteine levels and the difference between blood sampling periods. Maternal 5-MTHF levels significantly decreased and total homocysteine significantly increased from early pregnancy to birth as the pregnancy advanced. Maternal FA levels were significantly decreased at delivery compared to early pregnancy. At birth, cord 5-MTHF levels were much higher than maternal levels, while FA levels did not differ between these samples. Cord total homocysteine levels were lower than those in the mother.

**Figure 2:**
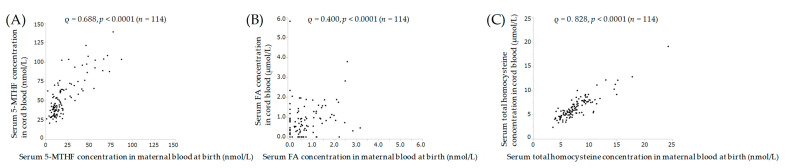
Correlation of 5-MTHF (**A**), FA (**B**) and (**C**) total homocysteine between maternal and cord blood at birth. Spearman correlation coefficient ρ, and *p*-value. 5-MTHF, 5-methyltetrahydrofolate; FA, folic acid.

Figure 2 shows the results of correlations between maternal blood and cord blood at delivery. Serum levels of 5-MTHF, FA and total homocysteine showed significant positive correlations between maternal and cord blood. 

Table 4 shows the correlation between homocysteine and 5-MTHF and FA at each blood sampling period (early pregnancy, late pregnancy, at birth, and cord blood). A significant negative correlation was seen between 5-MTHF to total homocysteine level at all sampling periods, but no significant correlation with FA was identified.

## 4. Discussion

In this study, longitudinal evaluation of 5-MTHF, FA and homocysteine in the serum of maternal and cord blood was performed on Japanese pregnant women. It was found that 5-MTHF levels decreased as gestation progressed, whereas serum FA levels were slightly decreased only at delivery compared to early pregnancy. A cross-sectional analysis showed a significant negative association between 5-MTHF and total homocysteine at all sampling periods, but no relationship between FA and homocysteine. At delivery, cord 5-MTHF levels were much higher than maternal levels, while no significant difference was seen in FA.

Governments such as those in North and South America enforce a policy of mandatory FA fortification for grain products. However, no such policy has yet been adopted in Japan. In the present study, median maternal 5-MTHF levels during pregnancy were 14.1–32.2 nmol/L, and median FA levels were 0.433–0.620 nmol/L. In Germany, median maternal serum 5-MTHF levels (10–90th percentiles) were 15 (4.0–41.9) nmol/L by LC-MS/MS [19], close to the results of our study. Germany does not enforce FA fortification, and pregnant women are encouraged to voluntarily take FA supplements [19]. The results of that study might thus be attributable to a similar environment to Japan. On the other hand, in reports from populations where mandatory FA has been fortified, mean [95% confidence interval (CI)] plasma FA at 13 weeks of gestation was 2.41 (1.99–2.88) nmol/L by LC-MS/MS [23], mean plasma 5-MTHF at 24 weeks of gestation was 39.2 ± 15.5 nmol/L by LC/MS [31] and 36.6 ± 16.3 nmol/L by LC/MS [25], median (95% CI) serum 5-MTHF at 27 weeks’ gestation was 65.3 (24.4–75.5) nmol/L by LC-MS/MS [32], and median (95% CI) serum FA was 0.92 (0.23–1.46) nmol/L by LC-MS/MS [32]. In an exceptional American study by Bodnar, median serum 5-MTHF (25–75th percentile) at gestational age 9.4 weeks for pregnant women with FA fortification was 34.4 (25.2–47.7) nmol/L by LC-MS/MS [20], a value close to that in our study. FA supplements are recommended for pregnant women in early pregnancy in Japan [33]. Furthermore, the proportion of FA intake during early pregnancy in this study was higher than that reported in a previous study of Japanese pregnant women [34,35,36], which may be why concentrations of 5-MTHF in the present study were close to those of Bodnar et al. Serum 5-MTHF and FA in Japanese pregnant women were mostly lower than those in populations from regions with mandatory FA fortification, due to the expected effects of FA exposure, as mentioned in previous studies [9].

Maternal blood 5-MTHF in this study decreased as gestational age progressed, whereas FA levels were slightly decreased only at delivery compared to early pregnancy. This might be due to a decrease in FA intake and rate of intake in late pregnancy compared to early pregnancy. In a previous study of pregnant women, total folate similarly decreased as gestational weeks progressed [37,38,39]. In addition, 5-MTHF is the major folate molecular species, accounting for 82%–93% of folate in blood, whereas FA constitutes only a small amount of total folate [40,41,42]. These results suggest that the longitudinal changes in total folate in previous studies were likely related to 5-MTHF. 

This study investigated the relationship of folate metabolism-related substances between mothers and cord blood, and found that both 5-MTHF and FA were significantly positively correlated between maternal and cord blood, cord 5-MTHF levels were much higher than those in maternal blood, while FA levels did not differ between them. Several previous studies have reported that cord blood 5-MTHF was similarly higher than that in maternal blood [19,25]. In addition, maternal FA may not actively accumulate to the fetus [23]. Three folate transporters have been found in placental syncytiotrophoblasts: folate receptor alpha, reduced folate carrier and heme carrier protein 1 [43,44]. The results of these studies thus suggest that FA might be transported to the fetus in a maternal blood-dependent manner, and 5-MTHF might be actively transported from mother to fetus against gradients in the placenta [19,25]. 

Total blood folate is known to be negatively associated with total homocysteine [8,45,46]. In the elderly (non-pregnant female) population in Germany, plasma 5-MTHF by LC-MS/MS and total homocysteine levels by gas chromatography–mass spectrometry showed a negative correlation [47], consistent with the present results. In our study, the relationship between serum 5-MTHF and total homocysteine levels was examined, and a significant negative correlation was disclosed, but no relationship was apparent between FA and total homocysteine. Therefore, 5-MTHF may reflect folate status during pregnancy.

This study had several limitations. First, the study included only one hospital-based population. Second, compared with the Japan Environment and Children’s Study (JECS) [48], a representative birth cohort study in Japan, maternal age was higher in the present study (mean, 31.2 ± 5.1 years), and smoking and drinking rates during pregnancy were lower than in the JECS, at 18.2% and 45.9%, respectively. Similarly, the distribution of household income was higher than in JECS. Folate status may be higher than a typical Japanese population, because of the influence of household income [49], alcohol consumption [31], and smoking habits [25,31]. Third, this study did not investigate blood during fasting. Previous studies have reported that blood FA and 5-MTHF levels are affected by the fasting state [50]. Fourth, this study did not investigate genetic polymorphisms affecting folate metabolism, such as methylenetetrahydrofolate reductase [51,52] and dihydrofolate reductase [13], which may affect the metabolism of 5-MTHF and FA. Fifth, this study used serum, and homocysteine values in serum are reportedly slightly higher than in plasma [6]. Re-methylation of homocysteine involves two methyl group transferring pathways, through 5-MTHF, using cofactor vitamin B12 and through betaine. In addition, there is a transsulfuration pathway for homocysteine. These related substances were thus not taken into account [6]. Finally, blood levels of 5-MTHF and FA in the blood of study subjects could not be evaluated in this study, because thresholds for excess and deficiency are unknown. In this study, FA was detected in the serum of Japanese pregnant women, and was considered to be unmetabolized FA, but no causal relationship between blood FA levels and negative fetal outcomes has been demonstrated. On the other hand, the benefits of FA in preventing NTD appear incontrovertible [53,54]. Further research is needed to establish optimal blood levels thresholds to balance NTD prevention with excess disease. In the future, it will be important to follow the children of the subjects of this study and to evaluate the relationship between FA overdose and health and disease outcomes in the children, using blood FA concentrations. On the other hand, blood levels of 5-MTHF should be considered as a more sensitive indicator of maternal folate deficiency. The accumulation of this information could provide evidence for appropriate FA use and public health policy.

## 5. Conclusions

The present results suggested that 5-MTHF was more likely to be transferred to the fetus than FA, correlated negatively with total homocysteine, and represents a physiologically important molecular species in folate metabolism that may reflect folate status during pregnancy. Further research is needed to establish optimal blood levels of 5-MTHF and FA for fetuses.

## Figures and Tables

**Table 1:** Characteristics of the mothers.

Variables	*n*	(%)	Mean (SD)
Age at delivery, years	130		32.3 (4.6)
<20	1	0.8	
20 to <24	5	3.8	
25 to <29	34	26.2	
30 to <34	48	36.9	
35 to <39	34	26.2	
≥40	8	6.2	
Missing	16	-	
Body mass index before pregnancy, kg/m^2^	130		21.8 (3.1)
<18.5	15	11.5	
18.5 to <25.0	97	74.6	
≥25	18	13.8	
Missing	16	-	
Marital status			
Married	129	99.2	
Unmarried	1	0.8	
Divorced/widowed	0	0.0	
Missing	16	-	
Parity	129		0.83 (0.78)
0	49	38.0	
1	56	43.4	
≥2	24	18.6	
Missing	17	-	
Smoking habits in late pregnancy			
Never smoked	110	90.9	
Ex-smokers who quit before pregnancy	9	7.4	
Smokers during late pregnancy	2	1.7	
Missing	25	-	
Alcohol consumption			
Never drank	117	97.5	
Drinkers during pregnancy	3	2.5	
Missing	26	-	
Household income, million Japanese-yen/year			
<2	0	0.0	
2 to <4	22	19.5	
4 to <6	34	30.1	
6 to <8	32	28.3	
8 to <10	15	13.3	
≥10	10	8.8	
Missing	33	-	
FA intake in early pregnancy, µg/day			
0	59	45.4	
>0	71	54.6	
>0 to <100	6	4.6	
100 to <200	3	2.3	
200 to <300	14	10.8	
300 to <400	7	5.4	
400 to <500	32	24.6	
500 to <600	3	2.3	
≥600	6	4.6	
Missing	16	-	
FA intake in late pregnancy, µg/day			
0	81	67.5	
>0	39	32.5	
0> to <100	3	2.5	
100 to <200	6	5.0	
200 to <300	5	4.2	
300 to <400	0	0.0	
400 to <500	23	19.2	
500 to <600	2	1.7	
≥600	0	0.0	
Missing	26	-	

*SD*, standard deviation; -, Percent is not calculated for missing values; FA, folic acid.

**Table 2:** Characteristics of neonates.

Variables	*n*	(%)	Mean (SD)
Gestational age at birth			
Total, weeks	120		39.5 (1.1)
Preterm births (<37 weeks)	2	1.7	
Term births (37 to <42 weeks)	118	98.3	
Postterm births (≥42 weeks)	0	0.0	
Missing	25	-	
Sex			
Male	57	48.3	
Female	61	51.7	
Missing	28	-	
Type of delivery			
Vaginal	94	87.9	
Caesarean	13	12.1	
Missing	39	-	
Birth weight, g	121		3155 (369)
Low birth weight, <2500 g	4	3.3	
Missing	25	-	
Birth length, cm	121		49.5 (2.5)
Missing	25	-	

*SD*, standard deviation; -, Percent is not calculated for missing values.

**Table 3:** Distribution of serum 5-MTHF, FA and total homocysteine levels and difference between blood sampling periods.

		Maternal Blood									Cord Blood (*n* = 121)		
		Early Pregnancy (*n* = 146)			Late Pregnancy (*n* = 131)			At Birth (*n* = 116)					
Analytes		Median	25th	75th	Median	25th	75th	Median	25th	75th	Median	25th	75th
5-MTHF	nmol/L	32.2 ^a^	20.3	52.8	17.0 ^b^	11.6	31.7	14.1 ^c^	9.8	23.2	44.7 ***	36.5	64.2
FA	nmol/L	0.620 ^a^	0.095	1.221	0.620	0.127	1.205	0.433 ^b^	0.000	1.052	0.530	0.000	1.043
Total homocysteine	µmol/L	5.38 ^a^	4.58	6.36	5.61 ^b^	4.74	6.96	7.16 ^c^	5.88	9.16	6.02 ***	5.01	7.75

Values are presented as the median with 25th and 75th percentiles. Different letters indicate statistically significant differences between early and late pregnancy, and at birth. (Wilcoxon signed-rank test with Bonferroni correction, *p* < 0.0167 (*n* = 113)). *** *p* < 0.0001 Wilcoxon signed-rank test, maternal blood at birth vs. cord blood (*n* = 114). 5-MTHF, 5-methyltetrahydrofolate; FA, folic acid.

**Table 4:** Correlation of 5-MTHF or FA to total homocysteine in each blood sampling period.

Variable		5-MTHF		FA	
Blood Sampling Period	*n*	ρ	*p*-Value	ρ	*p*-Value
Early pregnancy	146	−0.356	<0.0001	−0.122	0.142
Late pregnancy	131	−0.518	<0.0001	−0.148	0.093
At birth	116	−0.544	<0.0001	−0.149	0.111
Cord blood	121	−0.394	<0.0001	−0.049	0.590

Spearman correlation coefficient ρ, and *p*-value. 5-MTHF, 5-methyltetrahydrofolate; FA, folic acid.
